# The Combined Use of Orf Virus and PAK4 Inhibitor Exerts Anti-tumor Effect in Breast Cancer

**DOI:** 10.3389/fmicb.2022.845259

**Published:** 2022-03-23

**Authors:** Hao Deng, Bin Xiao, Yinger Huang, Kongyan Weng, Jialing Chen, Kun Li, Hongfeng Wu, Shuhong Luo, Wenbo Hao

**Affiliations:** ^1^Institute of Antibody Engineering, School of Laboratory Medicine and Biotechnology, Southern Medical University, Guangzhou, China; ^2^Key Laboratory of Antibody Engineering of Guangdong Higher Education Institutes, Southern Medical University, Guangzhou, China; ^3^Department of Laboratory Medicine, The Sixth Affiliated Hospital of Guangzhou Medical University, Qingyuan People’s Hospital, Qingyuan, China; ^4^Department of Transfusion Medicine, Fujian Provincial Hospital, Shengli Clinical Medical College of Fujian Medical University, Fuzhou, China; ^5^Department of Laboratory Medicine, School of Medicine, Foshan University, Foshan, China; ^6^Guangdong Provincial Key Laboratory of Construction and Detection in Tissue Engineering, Southern Medical University, Guangzhou, China

**Keywords:** Orf virus, oncolytic virus, breast cancer, PAK4 inhibitor, cancer therapy

## Abstract

The parapoxvirus Orf virus (ORFV) has long been recognized as one of the valuable vectors in researches of oncolytic virus. In order to develop a potential therapeutic strategy for breast cancer based on the oncolytic virotherapy *via* ORFV, firstly we explore the oncolytic effects of ORFV. Our research showed that ORFV exerts anti-tumor effects *in vitro* by inducing breast cancer cell G2/M phase arrest and cell apoptosis. *In vivo* experiments were carried out, in which we treated 4T1 tumor-bearing BALB/C mice *via* intratumoral injection of ORFV. ORFV can exert anti-tumor activity by regulating tumor microenvironment (TME) and inducing a host immune response plus directly oncolytic effect. The CRISPR-Cas9 knockout library targeting 507 kinases was used to screen out PAK4, which is beneficial to the anti-tumor effect of ORFV on breast cancer cells. PF-3758309 is a potent PAK4-targeted inhibitor. Co-using of ORFV and PF-3758309 as a combination treatment produces its anti-tumor effects through inhibition of cell viability, induction of apoptosis and suppression of cell migration and invasion *in vitro*. The results of *in vivo* experiments showed that the tumor growth of mice in the combination treatment group was significantly inhibited, which proved that the combination treatment exerts an effective anti-tumor effect *in vivo*. In summary, we have clarified the oncolytic effect of ORFV on breast cancer, and found that the combination of ORFV and PAK4 inhibitor can effectively improve the oncolytic effect of ORFV. We hope our research could provide a new idea for the development of new treatment strategies for breast cancer.

## Introduction

Breast cancer is the most common cancer and the leading cause of cancer-related death in women worldwide (Bray et al., 2018). The traditional methods of breast cancer treatment include surgery, chemotherapy, radiotherapy and drug therapy (Vaidya et al., 2018). In recent years, the successes associated with immunotherapy as a cancer treatment have resulted in a major shift in both cancer research and clinical practice (Schirrmacher, 2019). A milestone in breast cancer treatment was the first HER2-targeted therapeutic monoclonal antibody, named trastuzumab (trade name, Herceptin ^®^), approved by the FDA for treatment of HER2-positive breast cancer (Tolaney et al., 2015). However, the limitation of trastuzumab treatment is that it is only effective in HER2-positive patients, and the reality of single-agent immunotherapies is that many patients will not experience long-term durable benefits (La-Beck et al., 2015; Pera et al., 2016). Therefore, there is a need to develop new therapeutic modalities to overcome such limitations.

Oncolytic virus (OVs), an ideal therapeutic platform, are currently regarded as a potential treatment option for cancer patients who have low survival rates or limited treatment options. The anti-tumor activity of oncolytic viruses has previously been reported in different types of cancer in *in vivo* and *in vitro* studies. As a member of poxviridae, Orf virus (ORFV) is a potential candidate for oncolytic therapy because of its unique immune activation ability (Ning et al., 2013; Chen et al., 2017; Wang et al., 2019). Our team identified NA1/11, a strain of ORFV isolated from Jilin Province of China (Li et al., 2012), as a novel OV capable of inducing anti-tumor effect in colorectal cancer (CRC). The anti-tumor effect has been proven through *in vitro* and *in vivo* experiments. ORFV can infect and replicate in CRC and reduce lung metastasis of CRC (Chen et al., 2020). Direct cell lysis has long been identified as the mechanism by which OVs kill tumor cells. In the most recent years, more and more studies have reported that OVs can regulate tumor microenvironment (TME) and activate innate and adaptive anti-tumor immune responses (Bartlett et al., 2013; Hwang et al., 2020).

Some researchers have proposed another strategy to combine oncolytic viruses with other tumor immunotherapies. At present, a number of preclinical research data have confirmed that this strategy can achieve better efficacy in a variety of tumor animal models (Martin and Bell, 2018; Ribas et al., 2018). The use of the CRISPR/Cas 9 system to construct genomic libraries has made great progress in the application of high-throughput screening and functional genomics, which has become one of the main strategies for studying diseases, especially tumor target genes (Zhou et al., 2014; Manguso et al., 2017; Xue et al., 2020). This technology also provides a new idea for oncolytic viruses to screen out suitable targets for action or drug combination targets. We screened PAK4 as a potential target to enhance the oncolytic effect of ORFV. PAK4 is a kinase involved in a wide range of biological activities, including protecting cells from apoptosis, inhibiting cell adhesion, promoting cell migration, and anchor independent growth. High expression of PAK4 is usually associated with poor prognosis of tumors (Costa et al., 2019). PF-3758309 is an orally available, potent and reversible PAK4 ATP-competitive inhibitor, which has been reported that it can inhibit tumor cell anchorage-independent growth, induce tumor cell apoptosis, and inhibit tumor cell proliferation (Murray et al., 2010). As evaluated by *in vitro* and *in vivo* experiments, PF-3758309 is expected to be used as a drug for ORFV combination therapy.

In this study, we evaluated the anti-tumor effects of an oncolytic virus, ORFV, in both human and murine breast cancer cell lines. ORFV reduces the growth of breast cancer cells by regulating cell cycle and apoptosis, which can be proved by *in vitro* experiments. The oncolytic effect of ORFV, which has been demonstrated through *in vivo* experiments, depends not only on the virus-induced tumor apoptosis, but also on the induction of host anti-tumor immune response. We screened out candidate kinases that affect the oncolysis of ORFV by using the kinase knockout library cells constructed by the CRISPR/Cas 9 system. Through siRNA verification experiment and bioinformatics analysis, PAK4 was screened out as a related kinase that could enhance the anti-tumor effect of ORFV. It has also been determined through *in vitro* and *in vivo* experiments that PF-3758309, the small molecule inhibitor of PAK4, can enhance the oncolytic effect of ORFV. This work will provide fresh insight that the combination of ORFV and PAK4 inhibitors is expected to be a new approach for breast cancer anti-tumor therapy.

## Materials and Methods

### Cell Lines and Virus

Human breast cancer cell lines (MCF-7 and MDA-MB-231) and murine breast cancer cell line 4T1 were purchased from the cell bank of the Chinese Academy of Sciences (Shanghai, China). The MCF-7 kinase knockout library cells, used CRISPR-Cas9 technology to target 507 kinases, were purchased from Focus Biology (Nanchang, China). All cells were cultured at 37°C in DMEM supplemented with 10% fetal bovine serum (FBS, Biological Industries, Beit-Haemek, Israel). We have successfully isolated and obtained Orf virus (NA1/11 strain). And the method of virus propagation and purification refers to our team’s previous research (Li et al., 2012).

### Reagents

PAK4 inhibitor PF-3758309 was purchased from Selleck Chemicals (Shanghai, China). In the *in vitro* experiment, the mother liquor of PAK4 inhibitor was dissolved in DMSO and diluted with the basic medium. In the *in vivo* experiment, it is dissolved by the ratio of 5% DMSO + 30% PEG300 + 65% normal saline (Murray et al., 2010; Bradshaw-Pierce et al., 2013; Pitts et al., 2013).

### Reverse Transcription-Quantitative (RT-q) PCR

Total RNA was extracted through Cell Total RNA Isolation Kit (FOREGENE, Chengdu, China) and reverse transcribed to cDNA using a reverse transcription kit (TOYOBO, Shiga, Japan). Quantitative real-time PCR was carried out with SYBR master mixture (TOYOBO, Shiga, Japan) on ABI 7500 Real-Time PCR platform following the manufacturer’s instructions (Thermo Fisher Scientific, MA, United States). We use a 3-step cycle protocol which was constructed following Toyobo’s guidelines: 1. Pre-denaturation in 95°C for 1 min; 2. 40 cycles were performed, denaturation in 95°C for 15 s, annealing in 65°C for 15 s and extension in 72°C for 45 s. The primers used were shown in Table 1. The results were presented as the mean ± SEM from three experiments.

**TABLE 1:** Primer list.

Target	Primer	Primer sequence (5′–3′)
CRISPR/Cas9	Sense	TCTTGTGGAAAGGACGAAACACCG
	Antisense	CTTCTCTAGGCACCGGATCAATTGC
CCL3	Sense	CTGCCCTTGCTGTTCT
	Antisense	AAGGCTGCTGGTTTCA
CCL5	Sense	ACCACTCCCTGCTGCTT
	Antisense	TGATGTATTCTTGAACCCACT
CCL19	Sense	ACTGCTGCCTGTCTGTGA
	Antisense	AGGGCTGGTCTGGAGGT
CXCL12	Sense	CTGCCGGTTCTTCGA
	Antisense	CATCTTGAGCCTCTTGTTT
ORFV035	Sense	AGCAGACTATTTATTCGGGAGG
	Antisense	TGCGGACAAGAAGATGACG
β-tubulin	Sense	AACTGGGACGACATGGAGAAA
	Antisense	GGATAGCACAGCCTGGATAGCA

### Cell Viability Assay

Cells were prepared in 96-well plates at the concentration of 3 × 10^3^ cells/well. Cells were infected with ORFV (MOI = 0, 1, 2, 5) or treated with PF-3758309 (20 nM) for various time points. Cell viability was detected by CCK-8 assay using CCK-8 solution (Sangon Biotech, Shanghai, China) in accordance with the manufacturer’s instruction.

### Cell Colony Formation Assay

Colony forming ability of MDA-MB-231, MCF-7 and 4T1 cells infected with ORFV (MOI = 5) or PBS was evaluated by colony formation assay. The cells grown in log phase were seeded on a 6-well plate at a density of 3,000 per well. After ORFV treatment, cells were fixed with absolute ethanol and stained with 0.1% crystal violet solution. The experiments were repeated at least three times.

### Flow Cytometry Analysis of Apoptosis

The flow cytometry assay was performed to detect cell cycle and apoptosis of tumor cells. The breast cancer cells infected by ORFV (MOI = 5) or PBS for 24 h of detecting cell cycle, and undergone various treatments for 48 h of detecting apoptosis. The collected cells were processed using Cell Cycle Staining Kit and Annexin V-FITC/PI Apoptosis Detection Kit (Lianke Biotech, Hangzhou, China) according to the manufacturer’s instructions and detected using Guava Flow Cytometer (Millipore, MO, United States).

### Western Blot Analysis

Cells were lysed in 1 × SDS protein loading buffer to obtain a total protein sample. The proteins separated on a 12% SDS polyacrylamide gel and blotted on a PVDF membrane. After blocking the 5% skim milk powder at room temperature for 2 h, the primary antibody was incubated overnight at 4°C, the secondary antibody was incubated at room temperature for 1 h, and then the PVDF membrane was washed 5 times with TBST. And visualized with ECL chemiluminescence reagent (Yeasen, Shanghai, China) using GeneGnome Chemiluminescence Imaging System (Gene, Hong Kong, China).

### Orf Virus Treatment *in vivo*

Female BALB/c mice (Laboratory Animal Center of the Southern Medical University, Guangdong, China) were used in experiments at 6 weeks of age. All animal testing procedures were strictly in accordance with the regulations of the Laboratory Animal Care. In the allograft tumor model, 4T1 cells (10^6^ in 100μL PBS) were subcutaneously injected into BALB/c mice. When tumors average volume about 50 mm^3^ (tumor volume (mm^3^) = 0.5 × length × width^2^), mice were randomly divided into four groups: ORFV group was received ORFV (10^7^ pfu) *via* intratumoral injection and normal saline by oral gavage; PAK4i group was received PBS *via* intratumoral injection and PF-3758309 (20 nM) by oral gavage; Combo group was received ORFV (10^7^ pfu) *via* intratumoral injection and PF-3758309 (20 nM) by oral gavage; and Mock group was received PBS *via* intratumoral injection and normal saline by oral gavage. At the end of the experiment, mice were sacrificed by cervical dislocation. The organs and tumor tissues were isolated and fixed in 10% buffered formalin for subsequent experiments.

### Tumor Tissue Immunofluorescence and Immunohistochemistry

Tumor tissues and organs isolated from the mice were fixed and embedded in paraffin. Paraffin sections were made by Servicebio company (Wuhan, China). The slides of the tumor tissues were used for IHC staining of cleaved caspase 3 (Cell Signaling Technology, MA, United States), and IF staining of Ki67 (Proteintech, IL, United States).

### Immunological Testing of Mice

Three days after ORFV intratumoral injection treatment, the mice were sacrificed and the spleen and tumor tissues were isolated. HE staining and CD3 IHC staining of spleen were completed by Servicebio company (Wuhan, China). Total RNAs were extracted from the tumor tissues and subjected to qPCR to determine the expression of CCL3, CCL5, CCL19, and CXCL12.

### Screening Candidate Kinases

After 72 h of infection of MCF-7 kinase knockout library cells with ORFV (MOI = 5), the surviving cells were collected and re-cultured. Genomic DNA was extracted until the number of cells was sufficient. Genomic DNA of untreated MCF-7 kinase knockout library cells were extracted as a control.

Using the extracted genomic DNA as a template, PCR is performed with CRISPR/Cas 9 specific primers (Table 1) to obtain amplified products containing sgRNA specific sequences. The PCR amplified product was recovered and purified and sent to IGE Biotechnology (Guangzhou, China). for next-generation sequencing.

Design siRNAs corresponding to the candidate kinase, and use siRNA-NC as a control (Table 2). After 24 h of transfection with siRNA, MCF-7 cells were infected with ORFVGFP, genetically engineered ORFV carrying green fluorescence, for the next 24 h. Collect cells to detect green fluorescence by flow cytometry to estimate the infection efficiency of ORFV.

**TABLE 2:** siRNA list.

Target	Sequence (5′–3′)
siRNA-NC	UUCUCCGAACGUGUCACGUdTdT
DAPK1 siRNA	CGAGCUGUUUGACUUCUUAdTdT
SGK3 siRNA	GAGGUUUACUGUUUAUAAAdTdT
ULK3 siRNA	CGGAGAUUGAGAUCCUCAAdTdT
ERBB4 siRNA	GAGUCUAUGUAGACCAGAAdTdT
STK17B siRNA	GUUAGACAAUGUAUAUCAAdTdT
mTOR siRNA	GCUUCUAUGACCAACUGAAdTdT
MAPK1 siRNA	CGAGCAAAUGAAAGAUGUAdTdT
MST1R siRNA	GCGACUUUGACGUGAAGUAdTdT
PAK4 siRNA	GGCGCGAGCUGCUCUUCAAdTdT
ERBB3 siRNA	GUGGAUUCGAGAAGUGACAdTdT
SIK3 siRNA	CUAUCAAGAUCAUAGAUAAdTdT

### Transwell Invasion Assay

A Matrigel precoated chamber (Corning, MA, United States) was used to analyze cell invasion ability. Cells were exposed to ORFV or PF-3758309 or combined treatment for 24 h. The cells in serum-free medium are loaded into the upper chamber, and the lower chamber is loaded with medium containing FBS. After 24 h of incubation, non-invasive cells were removed, and invasive cells were stained with crystal violet. Finally, count the number of invaded cells through microscope.

### Statistical Analysis

Data are presented as mean ± SEM. Statistical analyses were performed with GraphPad PRISM (6.0) software. Significant differences between two groups were compared using a Student’s *t*-test (two-tailed). A one-way ANOVA was used in multiple comparisons. The Chou-Talalay method for drug combination is based on the median-effect equation, which provides the theoretical basis for the combination index (CI)-isobologram equation that allows quantitative determination of drug interactions, where CI < 1, = 1, and > 1 indicates synergism, additive effect and antagonism, respectively (Zhang et al., 2015). *P*-values of < 0.05 were considered significant, and *p < 0.05, **p < 0.01, and ***p < 0.001.

## Results

### Infection and Cytotoxic Activity of Orf Virus in Breast Cancer Cells

**FIGURE 1:**
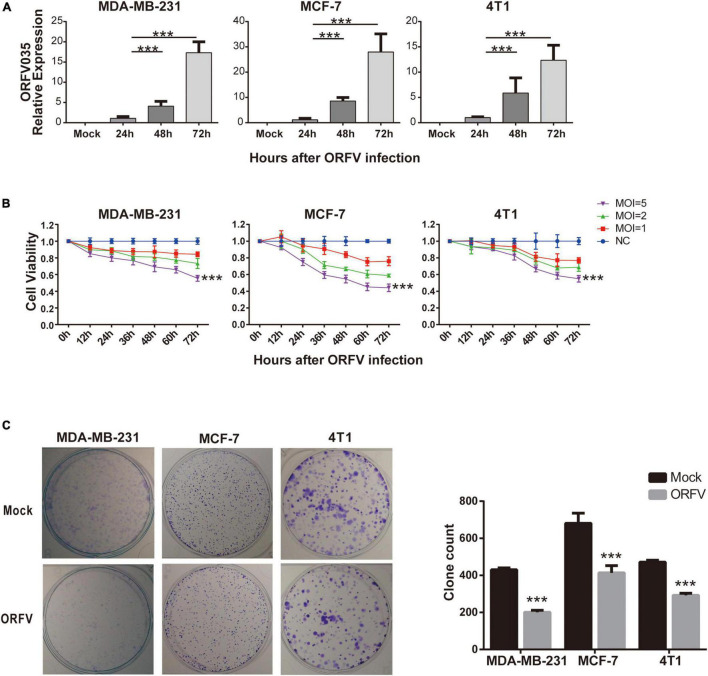
Infection and cytotoxic activity of ORFV in breast cancer cells. **(A)** MDA-MB-231, MCF-7 and 4T1 infected with ORFV at MOI of 1 for 0, 24, 48, and 72 h. The expression of ORFV035 gene was detected by qPCR. **(B)** The breast cancer cell lines (MDA-MB-231, MCF-7 and 4T1) were infected with ORFV at different MOI (0, 1, 2 and 5) for 0, 12, 24, 36, 48, 60, and 72 h. And cell viability was measured by CCK-8 assay. **(C)** Colony formation assay of MDA-MB-231 MCF-7 and 4T1 cells after infection with ORFV. The results are expressed as the means ± SEM of three independent experiments. ***p < 0.001.

We first infected three different breast cancer cells, including MDA-MB-231, MCF-7, and 4T1, with ORFV at MOI of 1. For the purpose of detecting ORFV replication, we compared the mRNA expression levels of ORFV core gene ORFV035 at different time points after ORFV infection (Figure 1A). The mRNA expression of ORFV035 increases in a time-dependent, suggesting that ORFV can replicate in breast cancer cells and reach a peak at 72 h. All three types of breast cancer cells have similar results, indicating that ORFV has no limitation on the type of tumor. We also observed the replication and packaging of virus particles in the cytoplasm through a transmission electron microscope (Supplementary Figure 1).

Different MOI (MOI = 0, 1, 2, 5) of ORFV infected three different breast cancer cells, and cell viability were detected through CCK-8 reagent at different time points. As the infection time increases, ORFV can effectively inhibit cell viability. In addition, ORFV with high MOI (MOI = 5) have the most significant inhibitory effect on cell viability (Figure 1B). And we found that after infection of ORFV with high MOI, the clonal formation ability will be effectively inhibited (Figure 1C). Which indicated the long-term inhibitory effect of ORFV on cell viability. On the contrary, even the high MOI ORFV does not have a growth inhibitory effect on normal cells (Supplementary Figure 2).

### Orf Virus Regulates Breast Cancer Cell Cycle and Apoptosis

**FIGURE 2:**
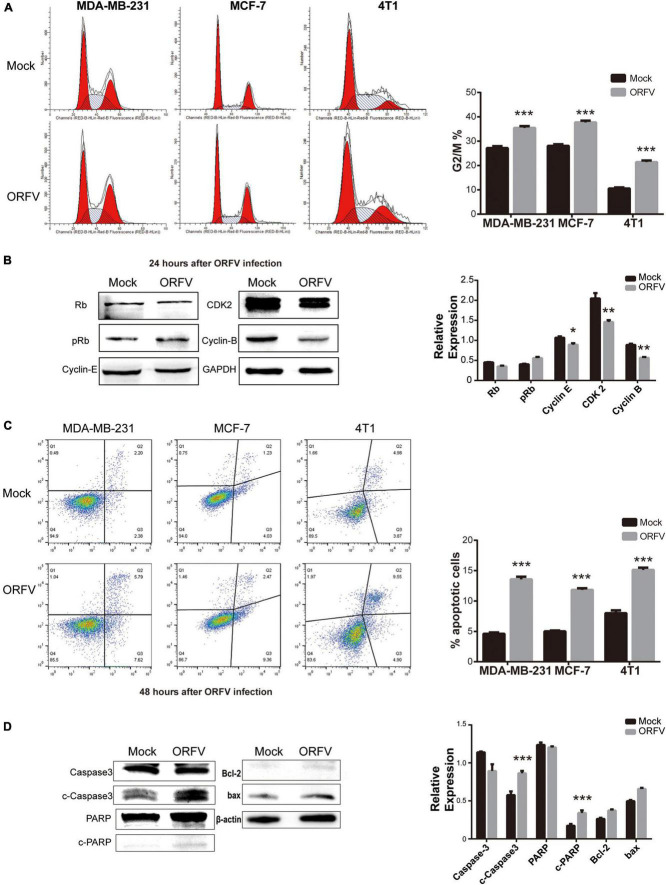
ORFV regulates breast cancer cell cycle and apoptosis. **(A)** MDA-MB-231, MCF-7 and 4T1 were infected with ORFV (MOI = 5) or PBS for 24 h. Flow cytometry detected cell cycle by Cell Cycle Staining Kit. **(B)** MDA-MB-231 cells were infected with ORFV (MOI = 5) or PBS for 24 h. Western blotting analyses of the relative expression of Rb, phospho-Rb (p-Rb), Cyclin-E, CDK2, Cyclin-B. **(C)** MDA-MB-231, MCF-7 and 4T1 were infected with ORFV (MOI = 5) or PBS for 48 h. Flow cytometry detected apoptosis by Annexin V-FITC/PI Apoptosis Detection Kit. **(D)** MDA-MB-231 cells were infected with ORFV (MOI = 5) or PBS for 48 h. Western blotting analyses of the relative expression of cleaved Caspase-3 (c-Caspase-3), cleaved PARP (c-PARP), Bcl-2 and bax. The results are expressed as the means ± SEM of three independent experiments. *p < 0.05; **p < 0.01; ***p < 0.001.

In order to investigate the mechanisms of ORFV inhibiting cell growth, we evaluated the changes in the cell cycle progression of breast cancer cells using flow cytometry. We observed that the cell cycle of MDA-MB-231, MCF-7, and 4T1 infected by ORFV will be blocked in the G2/M phase, which means that the mitotic process of cells is inhibited (Figure 2A). Furthermore, the western blot results demonstrated the change of cell cycle after MDA-MB-231 infection with ORFV for 24 h. It can be observed that cyclin B was significantly reduced while CDK2 and cyclin E were slightly decrease. The changes in Rb and p-Rb are not significant (Figure 2B).

The flow cytometry used to detect the effect of ORFV-induced apoptosis in breast cancer cells. The results showed that the number of total apoptotic cells was significantly higher in cells infected with ORFV for 48 h (Figure 2C). ORFV infection of MDA-MB-231 cells for 48 h led to a significant stimulation of caspase-3 and PARP activity. But there is no significant difference in the ratio of Bcl-2/bax protein expression (Figure 2D). We speculate that ORFV may play a role in the downstream of apoptosis.

### Orf Virus Inhibited Tumor Growth on 4T1 Allograft Tumor in BLAB/c Mice

**FIGURE 3:**
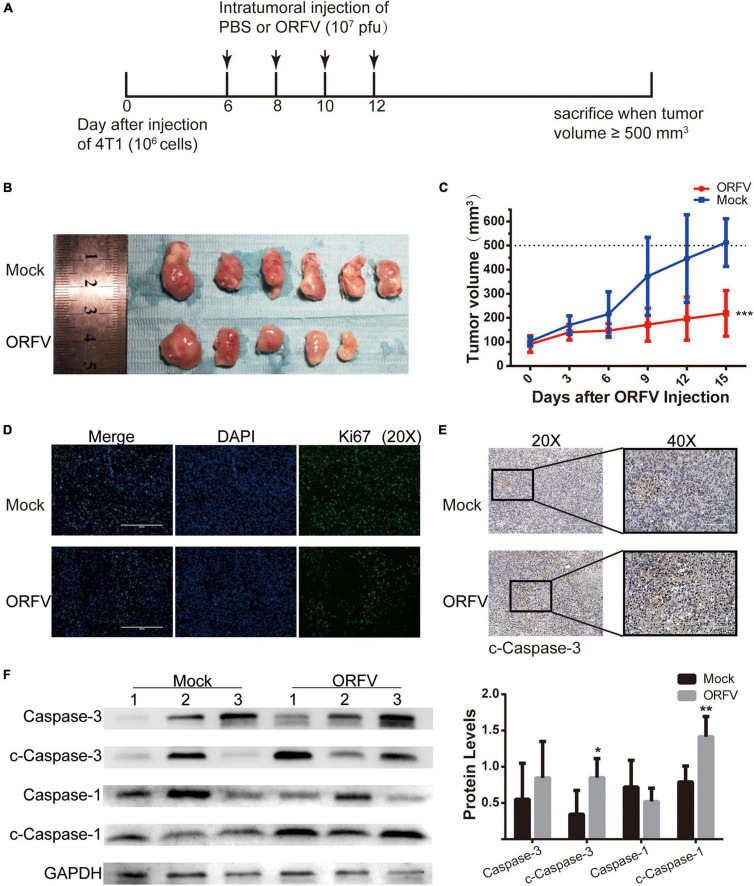
ORFV inhibited tumor growth on 4T1 allograft tumor in BLAB/c mice. **(A)** ORFV treatments on tumor-bearing mice was performed in accordance with the illustrated time schedule. **(B)** Images of 4T1 tumor of BLAB/c mice treated with PBS or ORFV. **(C)** The tumor volume was measured every 3 days, and the tumor growth curve was drawn. **(D)** Representative immunofluorescence results of Ki67 expression in tumor tissues of BALB/c mice treated with PBS or ORFV. **(E)** Representative IHC results of c-Caspase-3 expression in tumor tissues of BALB/c mice treated with PBS or ORFV. **(F)** Western blotting analyses of c-Caspase-3 and c-Caspase-1 in tumor tissues of BALB/c mice treated with PBS or ORFV. Mock1, 2 and 3 represent three of the mice in the Mock group. ORFV1, 2 and 3 represent three of the mice in the Mock group. The results are expressed as the means ± SEM of three independent experiments. *p < 0.05; **p < 0.01; ***p < 0.001.

We have verified that ORFV treatment does not produce serious toxicity *in vivo* experiments on nude mice (Supplementary Figure 3). Some studies have reported that OVs can regulate the TME and activate the innate and adaptive anti-tumor immune response. Then we investigated the anti-tumor effect of ORFV in 4T1 tumor-bearing mice with normal immunity. The animal treatment protocol is shown in Figure 3A. We found that tumor growth can be significantly repressed after intratumoral injection of ORFV (Figures 3B,C). The result was confirmed by immunofluorescence. As shown in Figure 3D, a significantly reduced in the number of Ki67-positive cells in the ORFV group (intratumoral injection of ORFV) in comparison with the Mock group. The c-caspase3-positive cells in the ORFV group were significantly higher than those in the Mock group, which proved that ORFV exerted oncolytic effects by inducing apoptosis (Figure 3E). As expected, western blot results showed that expression of c-Caspase-3 increased in tumor tissues after ORFV injection. In addition, we also detected the expression of c-Caspase-1, which is associated with apoptosis and inflammation. Its expression was significantly increased in ORFV-injected tumor tissues, suggesting that ORFV may cause an inflammatory response at the tumor site and change the tumor microenvironment (Figure 3F).

### Orf Virus Activates the Anti-tumor Effect of Host Immunity

**FIGURE 4:**
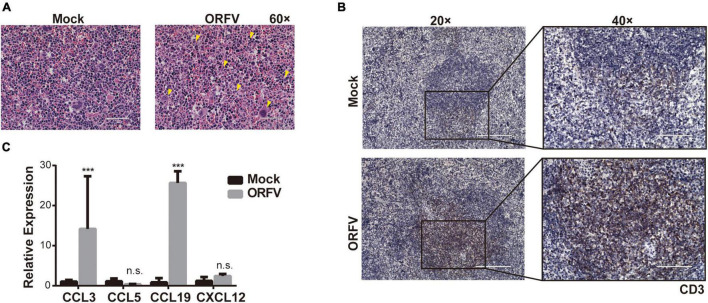
ORFV activates the anti-tumor effect of host immunity. **(A)** Representative images of the spleen by HE staining. Neutrophils and secondary follicles are represented by yellow triangular markers. **(B)** Representative IHC results of CD3 expression in spleen. **(C)** qPCR analyses of cytokines levels (CCL3, CCL5, CCL19, and CXCL12) in tumor tissue were treated with PBS or ORFV. The results are expressed as the means ± SEM of three independent experiments. n.s., *p* > 0.05; ***p < 0.001.

To determine whether ORFV could affect the immune system, we assessed the proportion of immune cells in the spleen. HE-staining results revealed that the phenotype of lymphocytes in the Mock group was normal. As compared with the Mock group, the spleen demarcation between the red pulp and white pulp was not clear in ORFV group. And we observed a significant increase in neutrophils and the appearance of secondary follicles (Figure 4A yellow triangular markers). Due to immune stimulation by antigens, primary lymphoid follicles, with concentrated chromosomes, sparse cytoplasm, and compact arrangement, will become secondary lymphoid follicles with larger cell size, with 1–3 small nucleoli distributed in the nuclear membrane edge. Increased numbers of neutrophils and secondary lymphoid follicles indicate activation of the body’s immune system. The T cell antigen receptor-CD3 complex plays an important role in the recognition and response to antigens. The IHC results showed that more CD3-positive cells appeared in the spleen of the ORFV group, which means that the body’s T cell immunity was activated (Figure 4B). Meanwhile, the intratumoral injection of ORFV changes the TME by up-regulating the chemokines CCL3 and CCL19, thereby activates the immune response (Figure 4C).

### Screening Out Candidate Kinases by Orf Virus Infection in Kinase Knockout Library Cells

**FIGURE 5:**
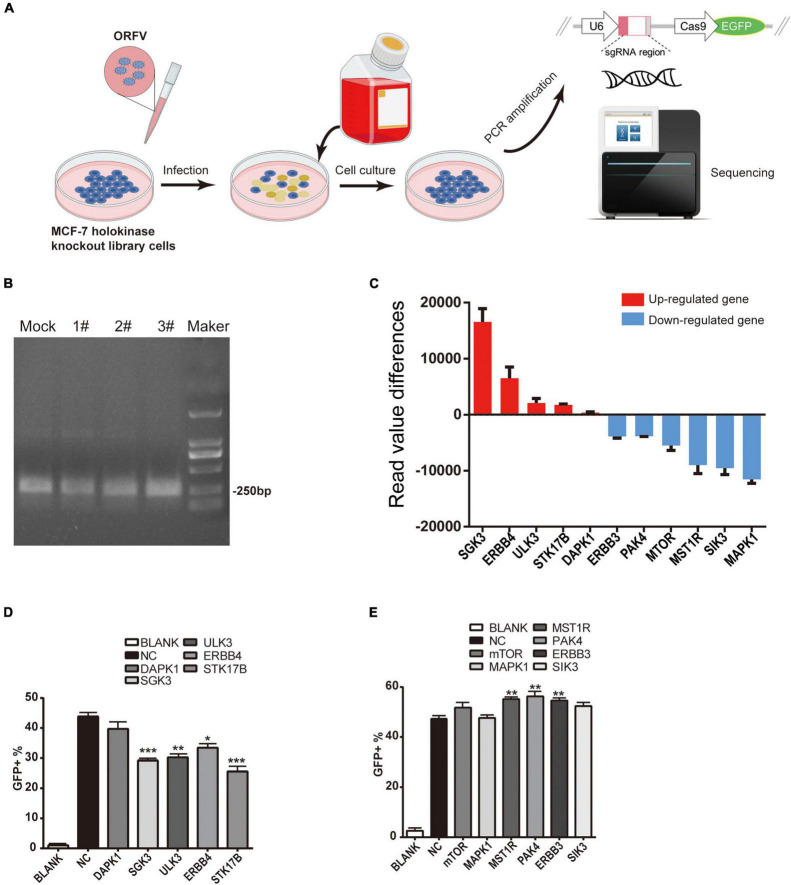
ORFV infectes CRISPR-Cas9 kinase knockout library cells to screen candidate kinases. **(A)** Schematic diagram of ORFV infection with CRISPR-Cas9 kinase knockout library cells to screen candidate kinases. **(B)** Use the specific primers to perform PCR amplification (The eluted solution is then used for next-generation sequencing. Mock represents control group PCR product; 1#, 2# and 3# represent ORFV group PCR product; Maker represent DL2000 marker). **(C)** The histogram shows the read value differences of the candidate kinases. The red histogram indicates that after gene knockout, the expression level of ORFV group is up-regulated compared to the control group. The blue histogram indicates that after gene knockout, the expression level of ORFV group was down-regulated compared with the control group. **(D)** Candidate kinases that inhibit ORFV infection after silencing by the corresponding siRNA. **(E)** Candidate kinases that enhance ORFV infection after silencing by the corresponding siRNA. The results are expressed as the means ± SEM of three independent experiments. **p < 0.01; ***p < 0.001.

The screening protocol is shown in Figure 5A. Specific primers were used for PCR, and the PCR products were recovered and purified and subjected to next-generation sequencing (Figure 5B). Results of candidate kinase screening are subjected to protein- protein interaction (PPI) network display, Kyoto Encyclopedia of Genes and Genomes Ontology (KO) pathway analysis and Gene Ontology (GO) analysis (Supplementary Data 1 and Supplementary Figure 4). We first screen the original sequencing data, sort the read values of the data, and finally screen out candidate kinases with higher read value differences (Figure 5C).

Then we designed some corresponding siRNAs for verification. After siRNA transfected MCF-7 cells for 24 h, add MOI = 1 ORFV with green fluorescent, continue to culture for 24 h. Set untreated MCF-7 cells as control. The proportion of cells infected by the virus was calculated through detecting GFP by flow cytometry. Flow cytometry results show that siRNA silencing the expression of SGK3, ULK3, ERBB4, STK17B can reduce the infection efficiency of ORFV-GFP virus (Figure 5D), while siRNA silencing the expression of MST1R, PAK4, ERBB3 can increase the infection of ORFV-GFP virus efficient (Figure 5E).

### Inhibition of PAK4 Can Enhance the Anti-tumor Effect of Orf Virus

**FIGURE 6:**
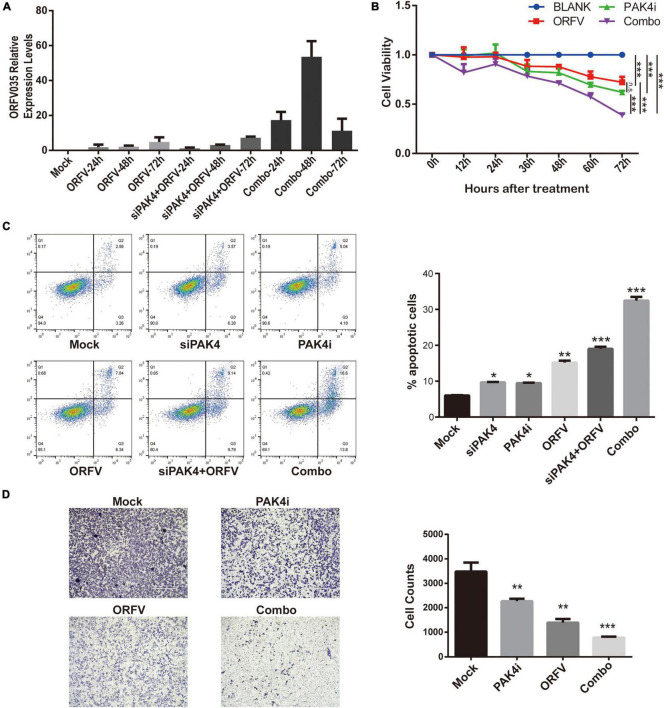
The inhibition of PAK4 can enhance the oncolytic effect of ORFV. **(A)** qPCR detection of the expression of ORFV035 after ORFV infection. **(B)** MCF-7 cells were infected with ORFV (MOI = 5) or treat with PAK4i (20 nM) or combine ORFV with PAK4i for 0, 12, 24, 36, 48, 60, and 72 h. And cell viability was measured by CCK-8 assay. **(C)** After MCF-7 undergoes various treatments for 48 h. Flow cytometry apoptosis by Annexin V-FITC/PI Apoptosis Detection Kit. **(D)** The invasion ability of MCF-7 cells was measured by transwell invasion assay. The results are expressed as the means ± SEM of three independent experiments. n.s., *p* > 0.05; *p < 0.05; **p < 0.01; ***p < 0.001.

PF-3758309 is the only ATP-competitive PAK inhibitor to have reached clinical trials (Murray et al., 2010). In this section, we investigate whether PAK4 inhibition with siRNA or PF-3758309 could enhance the oncolytic effects of ORFV. According to the grouping, cell samples were collected at 24, 48, and 72 h after ORFV treatment. Through reverse transcription and fluorescent quantitative PCR, it can be found that the mRNA expression level of PAK4 was down-regulated in both the siRNA group and the combination group after siRNA treatment (Supplementary Figure 5). We can also observe that there was no statistical difference in the expression of the viral core gene ORFV035 between the siPAK4 group and the ORFV group at the same time point after treatment (Supplementary Data 2, 3). Among them, Combo group showed that PF-3758309 is beneficial to the replication of ORFV (Figure 6A). The CCK-8 results showed that the Combo group not only showed a significant growth inhibitory effect on MCF-7, but also showed a stronger inhibitory ability compared to the single treatment group (Figure 6B).

The results of flow cytometry detection of cell apoptosis showed that transfection of siPAK4 itself did not cause apoptosis of host cells, and treatment of MCF-7 with 20 nM PF-3758309 for 48 h could induce partial cell apoptosis. The virus itself infects MCF-7 cells for 48 h to induce apoptosis in some cells, and the other two groups of combination groups have a more obvious effect of inducing cell apoptosis than the virus alone (Figure 6C). We designed a series of combined treatments and determine the therapeutic synergism using the Chou-Talalay method. The CIs of all treatment groups were < 1, suggested that PF-3758309 act synergistically with ORFV to enhance the anti-tumor effect in breast cancer (Supplementary Table 1 and Supplementary Figure 6).

We also conducted cell scratch test and transwell invasion assay. Although all three treatment groups can inhibit breast cancer cell migration, there is no significant difference between combo group and single treatment with ORFV or PF-3758309 (Supplementary Figure 7). The result in the transwell invasion assay showed that all three treatment groups can inhibit the ability of breast cancer cell invasion, and the combo group showed the most significant inhibitory ability. The above results indicate that PAK4 inhibitor can enhance the anti-tumor effect of ORFV on breast cancer (Figure 6D).

### PAK4 Inhibitors Increase the *in vivo* Oncolytic Effect of Orf Virus

**FIGURE 7:**
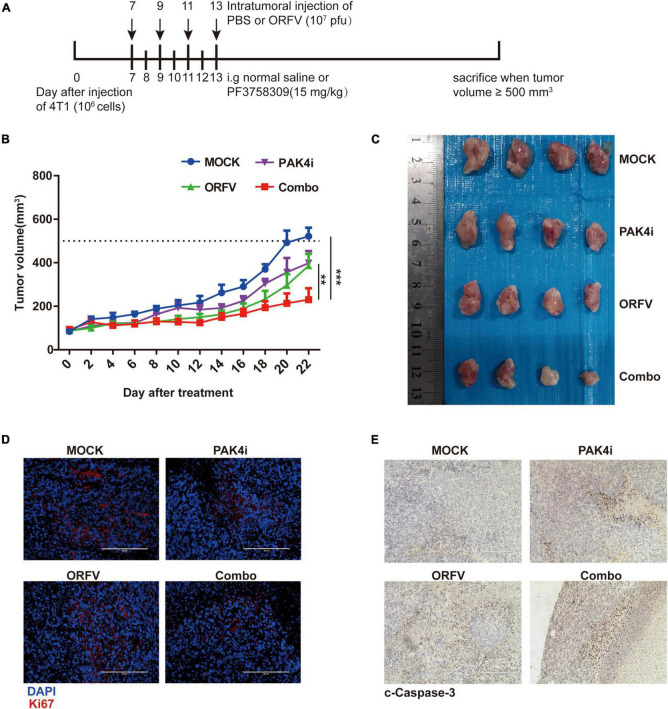
ORFV inhibited tumor growth on 4T1 allograft tumor in BLAB/c mice. **(A)** Different treatments on tumor-bearing mice was performed in accordance with the illustrated time schedule. **(B)** The tumor volume was measured every 2 days, and the tumor growth curve was drawn. **(C)** Images of 4T1 tumor of BLAB/c mice received various treatments. **(D)** Representative immunofluorescence results of Ki67 expression in tumor tissues of BALB/c mice received various treatments. **(E)** Representative IHC results of c-Caspase-3 expression in tumor tissues of BALB/c mice received various treatments. The results are expressed as the means ± SEM of three independent experiments. **p < 0.01; ***p < 0.001.

The animal treatment protocol is shown in Figure 7A. Drawing the tumor growth curve (Figure 7B), it can be observed that the tumors of the mice in the ORFV group and PAK4i group grew slower than the Mock group, and the Combo group showed significant tumor growth inhibition ability. When the tumor volume of the mice in the Mock group reached 500 mm3, the tumor sites of the mice were sampled and compared and analyzed. The tumor sizes of the mice in the three treatment groups were significantly different from those in the Mock group (Figure 7C). The Combo group showed the strongest anti-tumor effect.

Tumors with a high Ki67 positive rate are generally considered to grow faster and have a poor prognosis. Through immunofluorescence detection of tumors in mice, it can be observed that the number of Ki67-positive cells in the tumor site of the Combo group is the least (Figure 7D). The c-Caspase-3 protein is generally considered to be a marker protein for cell apoptosis. Through IHC detection of tumors in mice, it can be observed that the c-Caspase-3 positive cells in the tumor site of the Combo group are much higher than those of the Mock group (Figure 7E).

## Discussion

Since the twenty-first Century, the incidence rate and mortality rate of breast cancer have been increasing every year, which has become a huge potential threat to women health (DeSantis et al., 2019). Benefited from the rapid development and cross penetration of oncology, immunology, molecular biology and other disciplines, the research of tumor immunotherapy has developed rapidly showing great clinical efficacy and application prospects. As an emerging immunotherapeutic modality for cancer therapy, the oncolytic virus therapy has been widely recognized as a potential strategy to improve the clinical outcomes of breast cancer (Lawler et al., 2017; Harrington et al., 2019; Ahmed et al., 2020).

Researchers first put forward the concept of oncolytic virus in 1912 (Sinkovics and Horvath, 1993). In 2005, H101 oncolytic virus was first used in the treatment of head and neck tumors in China (Xia et al., 2004). Since the recent FDA approval of talimogene laherpaprevec (T-vec) in 2015 (Andtbacka et al., 2015; Johnson et al., 2015), oncolytic virus immunotherapy as a new therapeutic method has attracted more and more attention. A series of mammalian viruses and their genetically engineered forms have been classified as oncolytic viruses, including adenovirus (Hagihara et al., 2020), poxvirus (Chaurasiya et al., 2020), HSV-1 (Nakashima et al., 2018), Newcastle disease virus (NDV) (Burman et al., 2020), alphavirus (Liu et al., 2020), reovirus (Mahalingam et al., 2018) and so on. These oncolytic viruses share the common feature of the ability to selectively self-amplify in tumor cells and kill tumor cells without cytotoxicity to normal cells. With its unique biological characteristics, ORFV has the potential to become a new type of oncolytic viruses.

In this study, we first demonstrated that ORFV can specifically infect and replicate in breast cancer cells. ORFV can regulate breast cancer cell cycle to inhibit cell viability and induce cell apoptosis to directly kill tumor cells, reflecting the ability of anti-tumor effect. The results of cell cycle detection by flow cytometry and western blot showed that ORFV inhibits cell growth mainly by down-regulating cyclin B, CDK2 and cyclin E and regulating cell cycle arrest in G2/M phase. ORFV also can induce tumor cell apoptosis by activating Caspase-3 and cleaving PARP.

In addition, the safety of ORFV to normal cells and the selectivity to tumor cells were confirmed by *in vitro* experiments of normal cell line HUVEC infected with ORFV. The results of *in vivo* experiments in nude mice showed that the intratumoral injection of ORFV is safe for mice. ORFV does not induce additional cytotoxicity to the tissues at the injection site. At the same time, we drew the weight change curve of the mice and found no statistical difference in the weight change between the ORFV group and the control group. During the whole process of animal experiments, we also observed that the physiological state of mice did not produce any negative changes.

Growing evidence suggests that OVs are powerful stimulators of the host immune system (Gujar et al., 2011; Kaufman et al., 2015; Takasu et al., 2016; Brown et al., 2017). Tai et al. (2013) reported that ORFV can markedly increase immune cell activity in postoperative cancer surgery patients, which result in reduction of metastasis and recurrence of the cancer. Accordingly, we repeated ORFV treatment in tumor-bearing BALB/c mice with normal immunity, and the results shown that the ORFV treatment group can significantly inhibit tumor growth compared with the Mock group. As a tumor proliferation gene, Ki67 plays an important role in prognosis and prediction of breast cancer (Yerushalmi et al., 2010). It can be detected that the expression of Ki67 was decreased in the ORFV group in tumor tissues. On the contrary, the expression of c-Caspase3 was increased. The activation of caspase-3 in 4T1 is clearly correlated with the ORFV treatment, suggesting a direct oncolytic effect of ORFV mediated by inducing apoptosis. Up-regulation of CCL3 and CCL19 had been reported to stimulate innate and Th1 immune responses (Ravi et al., 2014; Qian et al., 2017). These results indicate that ORFV can induce tumor inflammation and TME change. Mouse spleen HE staining and IHC results are used to evaluate the activation of the body’s immune system after intratumoral injection of ORFV. These results showed that ORFV effectively activates the body’s immunity.

Recently, more and more clinical trials of OVs for cancer treatment have shown that small molecule inhibitors or immune checkpoint inhibitors (ICI) can increase virus specificity and enhance the oncolytic effect (Zamarin et al., 2014; Shen et al., 2016). Yan’s team also proved that the combination of PKA inhibitor and VCP inhibitor and M1 virus reduced the production of virus-induced PD-L1 + DC, MDSC, TAM and T-reg, thereby reduced tumor burden and improved the survival rate of mice, and eventually enhanced the oncolytic effect of M1 virus (Lin et al., 2014; Li et al., 2016; Zhang et al., 2017). The CRISPR/Cas9 technology was used for high-throughput screening of tumor target genes, which provided us with new ideas for screening the key kinases for ORFV to exert oncolytic effects (Pierik et al., 2021). We purchased the MCF-7 kinase knockout library cells constructed by CRISPR/Cas9, and infected the library cells with ORFV and used high-throughput sequencing to screen out a batch of candidate kinases related to the oncolysis of ORFV. Due to the interference of confounding information in the original sequencing data. We further filter the candidate kinases data by eliminating errors, irrelevant data, and data with read values less than 100. Subsequently, we ranked the valid data to obtain a list of candidate kinases. The corresponding siRNA were screened and designed to validate the candidate kinases. We also used online database to conduct bioinformatics analysis of the selected kinase PAK4. The result suggested that PAK4 kinase could be the target of ORFV combination therapy. Whether other kinases, such as MST1R, ERBB3, etc., have an impact on the anti-tumor effect of oncolytic viruses remains to be further studied by other members of the team.

In our experiments, we found out the infection and replication abilities of ORFV were not affected after the expression of PAK4 was silenced by using siRNA. As a competitive inhibitor of PAK4, the small molecule inhibitor PF-3758309 does not affect the expression of PAK4 in the host itself (Li et al., 2017; Chung et al., 2019). The combination of PF-3758309 and ORFV jointly exerted a stronger inhibitory effect on PAK4, as well as a stronger ability to induce cell apoptosis. Through detecting the expression level of the viral core gene ORFV035 in breast cancer cells, we found that the replication level of the virus seems to be greatly improved at 24 and 48 h after the combination treatment. However, the declined level of ORFV035 at 72 h could be explained by the death of the host cell and the completion of the virus replication and release. The results of cell scratch test and transwell invasion assay showed that the combination treatment can significantly inhibit the migration and invasion of breast cancer cells. The synergy determinations of combination treatment were conducted according to the Chou–Talalay method. The CIs of a series of combination treatments used in this study are all less than 1, suggested that PF-3758309 act synergistically with ORFV to enhance the anti-tumor effect in breast cancer. Whether PF-3758309 enhances the oncolytic effect of ORFV by simultaneously inhibit other proteins of the PAK family expect PAK4 still needs further research.

In this study, we use PF3758309 at a dose of 15 mg/kg and gavage once a day to maintain the blood concentration of the drug, which is half of the dose reported in the literature (Pan et al., 2017). We observed the tumor growth curves and found that the tumors of the mice in the ORFV group and the inhibitor group grew slower than the Mock group, and the effect of the combination treatment group showed the strongest inhibition ability of the tumor growth in all the groups. The tumor growth curves of the three treatment groups were statistically different from those of the Mock group. Whereas, the mechanism of the combined anti-tumor effect of ORFV and PAK4 inhibitors is still unclear. Therefore, more research is needed to confirm our conclusions. Collectively, our study reveals the therapeutic effectiveness of ORFV on breast cancer and highlights the potential application of ORFV in combination with PAK4 inhibitors in cancer treatment.

## Conclusion

In conclusion, the results drawn from previous experiences manifested that ORFV strain NA1/11 as an oncolytic virus cause anti-tumor effects by two major mechanisms. One is the direct tumor killing effect, including the regulation of cell cycle and the induction of apoptosis. The other one is that ORFV exerts an anti-tumor effect by improving the TME and consequently triggering anti-tumor immune response in host. Furthermore, the kinase knockout library cells were used to screen out PAK4 as a potential target of oncolytic virus. And we found that PF-3768309, the small molecule inhibitor of PAK4, combined with ORFV in the treatment of breast cancer can exert a stronger anti-tumor effect. Here, we provide some experimental evaluation of the combination treatment of ORFV and PAK4 inhibitors and our study could provide a new idea for the development of new treatment strategies for breast cancer.

## Data Availability Statement

The data analyzed in this study is subject to the following licenses/restrictions: Articles related to the sequencing results of CRISPR/Cas9 library cells after ORFV processing are yet to be published. Requests to access these datasets should be directed to HD, howarddax@163.com.

## Ethics Statement

The animal study was reviewed and approved by the Institutional Animal Care and Use Committee of Southern Medical University.

## Author Contributions

WH conceived and designed the project. WH and SL wrote the manuscript. HD, BX, YH, KW, JC, KL, and HW performed the experiments and bioinformatics analyses. All authors reviewed the manuscript.

## Conflict of Interest

The authors declare that the research was conducted in the absence of any commercial or financial relationships that could be construed as a potential conflict of interest.

## Publisher’s Note

All claims expressed in this article are solely those of the authors and do not necessarily represent those of their affiliated organizations, or those of the publisher, the editors and the reviewers. Any product that may be evaluated in this article, or claim that may be made by its manufacturer, is not guaranteed or endorsed by the publisher.

## Abbreviations

ORFV: Orf virus
OVs: Oncolytic viruses
TME: tumor microenvironment
CRISPR/cas9: clustered regularly interspaced short palindromic repeats/cas9
PAK4: p21-activated kinase 4.
