# Supplementary material for: The Combined Use of Orf Virus and PAK4 Inhibitor Exerts Anti-tumor Effect in Breast Cancer
Source: Front Microbiol. 2022 Mar 23;13:845259. doi: 10.3389/fmicb.2022.845259 (PMC8984157; doi:10.3389/fmicb.2022.845259)
Supplement: Supplementary file 4 [file Table_1.DOCX]

Supplementary Table 1. Combination of ORFV with PF-3758309 (PAK4i) against MCF-7 cell growth in CCK-8 assay in vitro^a^

| Compound | | Fractional Inhibition  (fa)^b^ | Parameters^c^ | | | CI^d^ |
| --- | --- | --- | --- | --- | --- | --- |
| ORFV  (MOI) | PAK4i  (nM) |  | m | Dm | r |  |
| 1 |  | 0.149 | m=0.32529, dm=300.408, r=0.83508 | | |  |
| 2 |  | 0.1383 |  |  |  |  |
| 5 |  | 0.2233 |  |  |  |  |
|  | 10.0 | 0.4723 | m= 0.14064, dm= 3.8719, r= 0.68014 | | |  |
|  | 20.0 | 0.4513 |  |  |  |  |
|  | 40.0 | 0.521 |  |  |  |  |
| 1.0 | 10.0 | 0.519 |  | | | 0.17457 |
| 1.0 | 20.0 | 0.535 |  |  |  | 0.22001 |
| 1.0 | 40.0 | 0.531 |  |  |  | 0.49071 |
| 2.0 | 10.0 | 0.55 |  |  |  | 0.07446 |
| 2.0 | 20.0 | 0.537 |  |  |  | 0.20996 |
| 2.0 | 40.0 | 0.624 |  |  |  | 0.03361 |
| 5.0 | 10.0 | 0.608 |  |  |  | 0.01734 |
| 5.0 | 20.0 | 0.594 |  |  |  | 0.04462 |
| 5.0 | 40.0 | 0.678 |  |  |  | 0.00761 |

^a^ Dose and effect data were obtained from the CCK-8 assay (average value of triplicate).

^b^ Fractional Inhibition represents the inhibition rate of MCF-7 cells treated with different agents. Fractional Inhibition (fa) = 1 - mean value of experimental wells / mean value of control group.

^c^ Parameters were calculated from the median-effect equation and the median-effect plot. M is slope, signifies shape; Dm is IC50 (ORFV in MOI, PAK4i in nM), signifies potency; and r is linear correlation coefficient, signifies conformity.

^d^ Combination index (CI) was calculated from the CI equation algorithms using CompuSyn software. CI=1, <1 and >1 indicates additive effect, synergism and antagonism, respectively.
