# Supplementary material for: The Combined Use of Orf Virus and PAK4 Inhibitor Exerts Anti-tumor Effect in Breast Cancer
Source: Front Microbiol. 2022 Mar 23;13:845259. doi: 10.3389/fmicb.2022.845259 (PMC8984157; doi:10.3389/fmicb.2022.845259)
Supplement: Supplementary file 5 [file Table_2.DOCX]

Supplementary Material

## Supplementary Figures


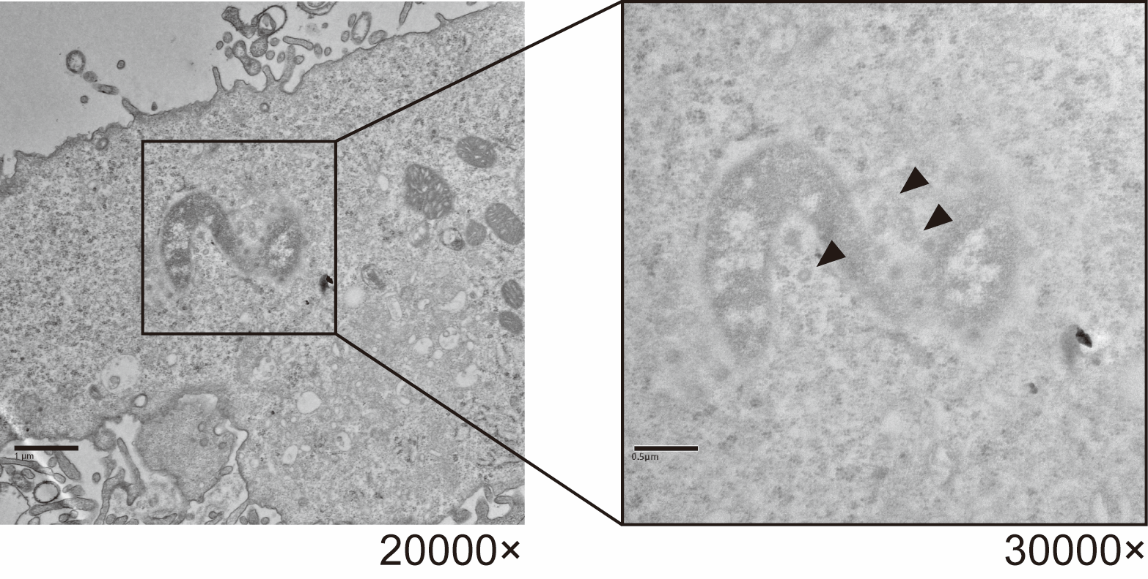


**Supplementary Figure 1.** ORFV replicates in breast cancer cells

4T1 cells were infected with ORFV at MOI of 1 for 48 hours. Image was obtained by transmission electron microscopy (TEM). The virus particles are marked with black triangle.

**
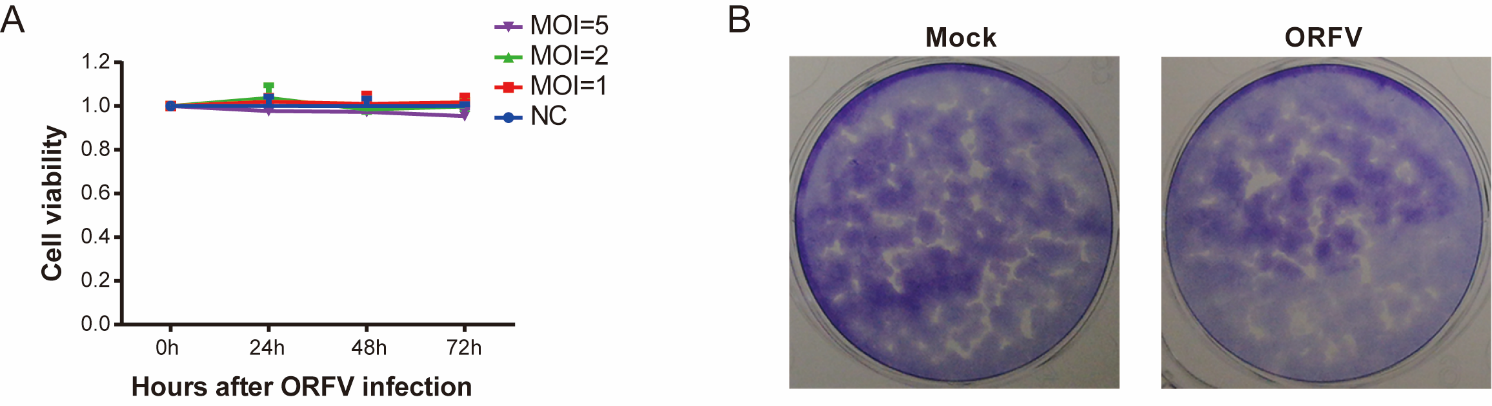
**

**Supplementary Figure 2.** ORFV did not affect normal cells

(A). HUVEC infected with ORFV at different MOI (0, 1, 2 and 5), and the cell viability was measured by CCK8 assay. (B). Colony formation assay of HUVEC treated with ORFV for 7 days.

**
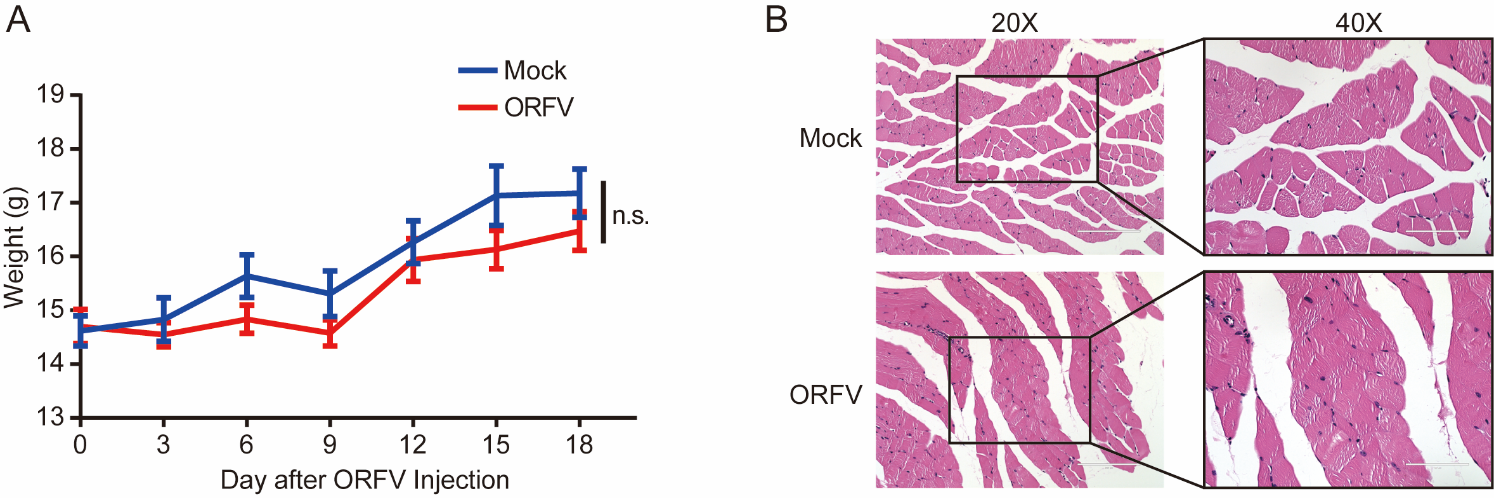
**

**Supplementary Figure 3.** Safety evaluation of ORFV in vivo treatment

(A). Body weight curve of nude mice after intratumoral injection of ORFV (n=5). There was no significant difference in body weight change between. (B). Representative image of tissue at the site of virus injection stained with HE. No pathological phenomena related to virus infection were found. The results are expressed as the means ± SEM. *, *p* < 0.05; **, *p* < 0.01; ***, *p* < 0.001


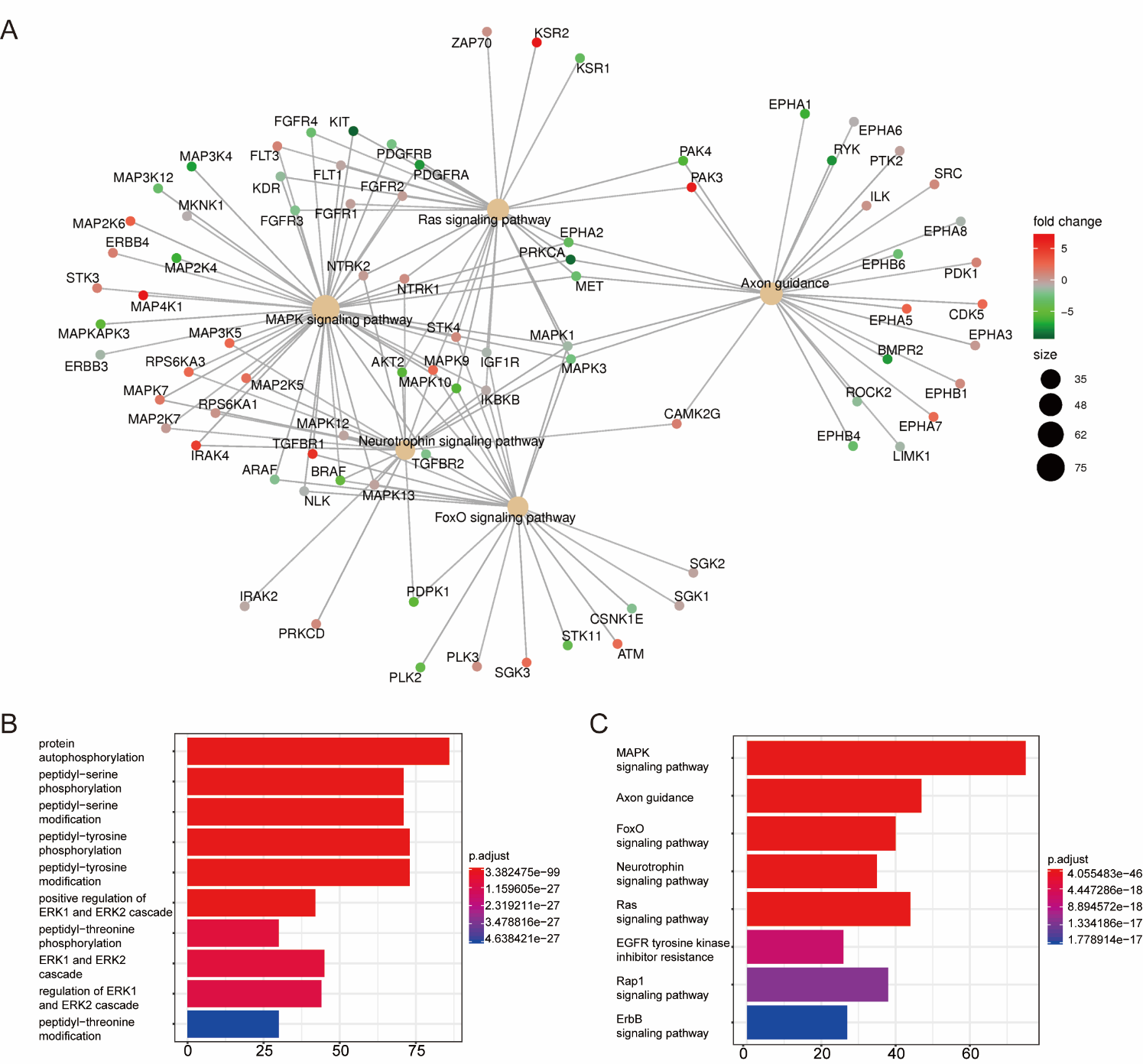


**Supplementary Figure 4.** Results of candidate kinases screening

(A) Schematic diagram of protein-protein interaction network in candidate kinases. (B) The enrichment result of the KO pathway in candidate kinases. D: The enrichment result of the Gene Ontology in candidate kinases.


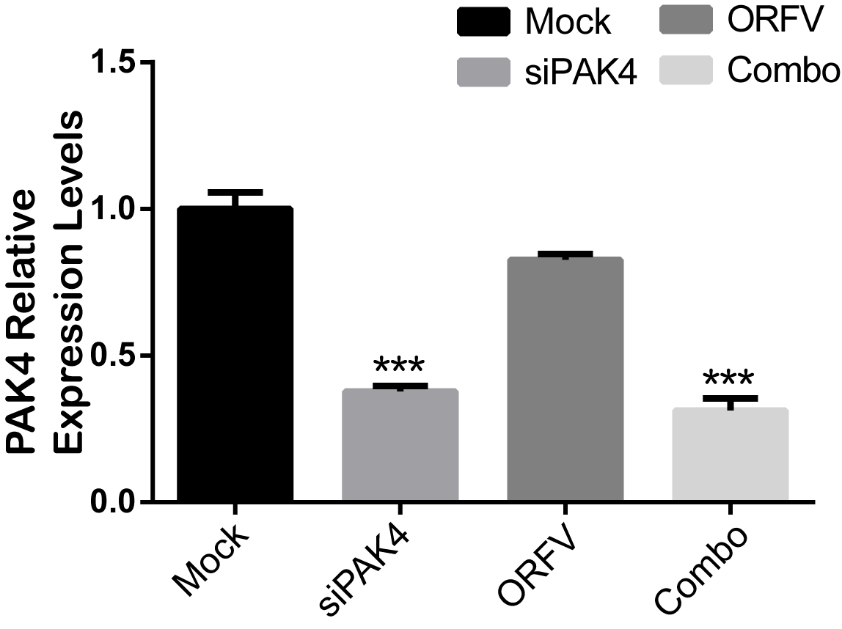


**Supplementary Figure 5.** Results of relative expression of PAK4

After 24 hours of transfection with siPAK4 or mock, MCF-7 cells were infected with ORFV (MOI=5). After 72 hours of infection, the relative expression of PAK4 was detected by qPCR.


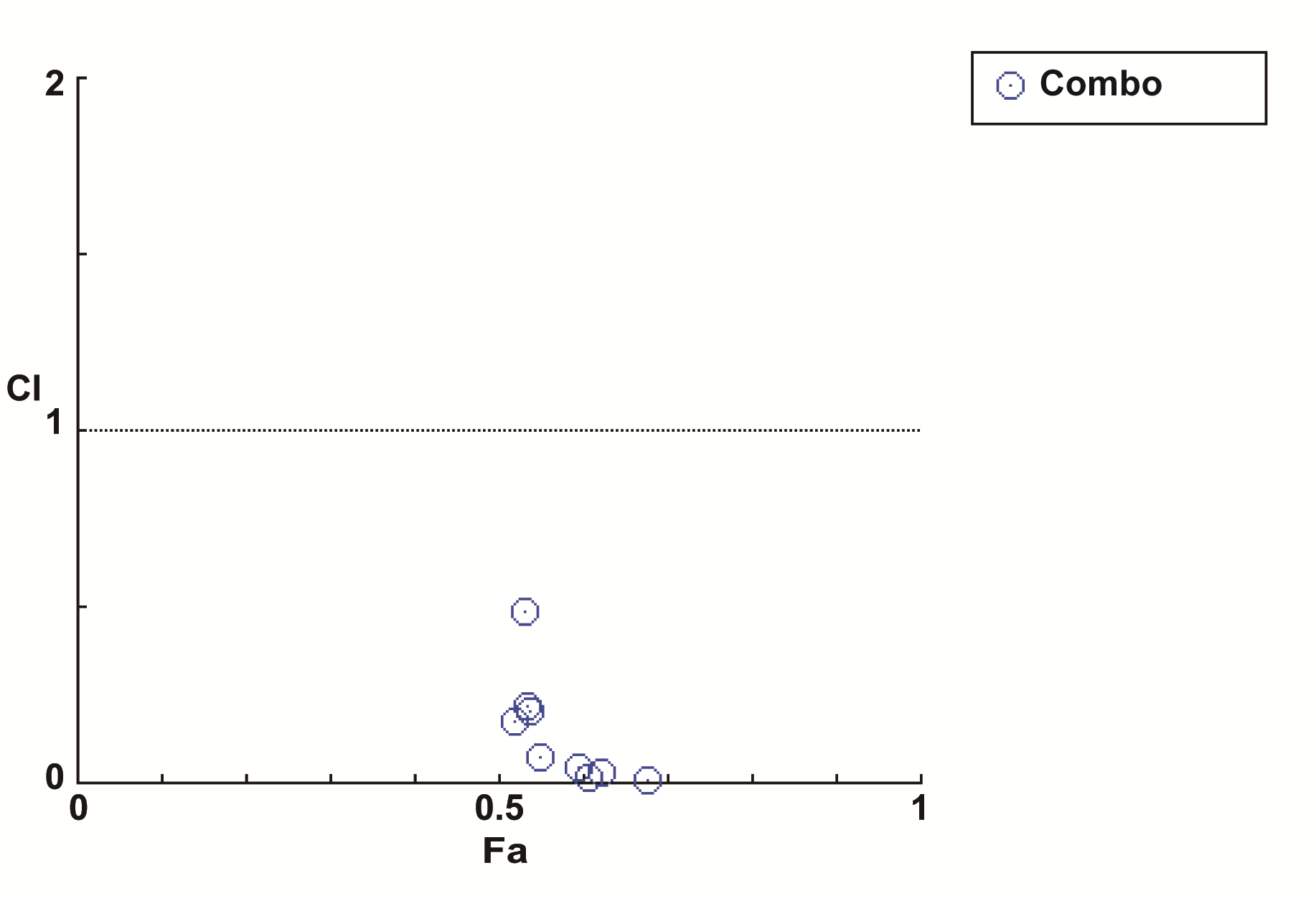


**Supplementary Figure 6.** The Combination index plot representations obtained for ORFV and PF3758309 combinations

MCF-7 cells treated with a series of combined treatments (ORFV, MOI=1/2/5; PAK4i, 10/20/40nM) for 72 hours. The Fractional Inhibition was obtained by CCK-8 assay. The graphic representations obtained from the CompuSyn Report.


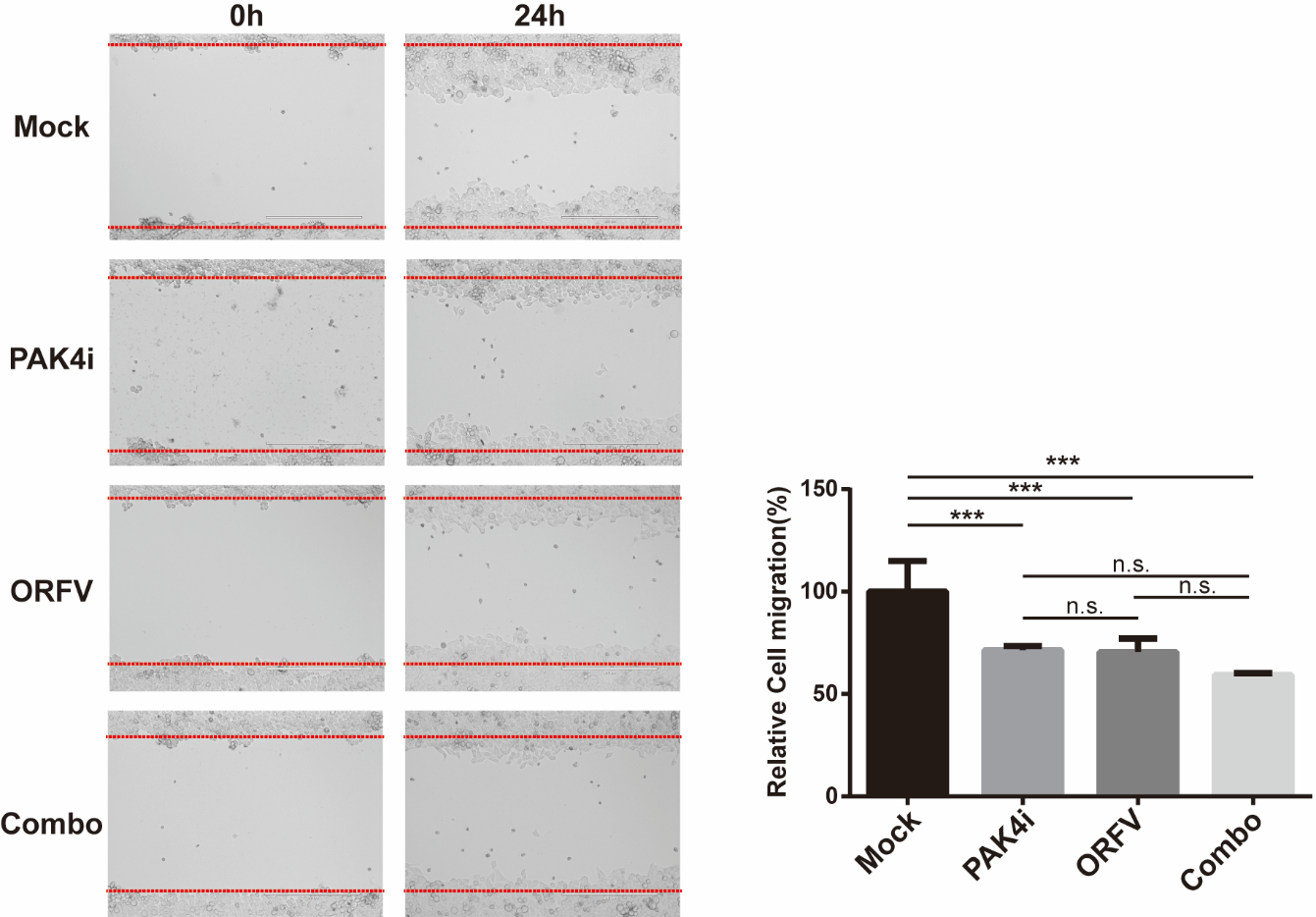


**Supplementary Figure 7.** Cell scratch test

MCF-7 Cells were seeded in 6-well plates at a density of 10^6^ cells per well and cultured for 24 hours. A wound was scratched in the cell lawn with a pipette tip and the cells were washed with serum-free DMEM three times to remove the debris. The cells were exposed to ORFV or PF-3758309 or combination treatment. Images of cell scratch test were captured at 24 hours. The results are expressed as the means ± SEM. *, *p* < 0.05; **, *p* < 0.01; ***, *p* < 0.001
